# Supplementary material for: Holy Basil (Ocimum sanctum L.) Flower and Fenofibrate Improve Lipid Profiles in Rats with Metabolic Dysfunction Associated Steatotic Liver Disease (MASLD): The Role of Choline Metabolism
Source: Plants (Basel). 2024 Dec 24;14(1):13. doi: 10.3390/plants14010013 (PMC11722946; doi:10.3390/plants14010013)
Supplement: Supplementary file 1 [file plants-14-00013-s001.zip › plants-3315767-supplementary.pdf]

SUPPLEMENTAL FIGURES

Figure S1. Caloric intake, and liver and kidney weights of rats fed either a normal diet (ND) or a high-fat diet (HFD). The rats were administered either vehicle (distilled water), *Ocimum sanctum* L. flower (OSLY) extracts at 250, 500, or 1,000 mg/kg body weight, and/or fenofibrate daily for 12 weeks. *Indicates significant difference (P<0.05) from the ND group; #indicates a significant difference (P<0.05) from the HFD group based on t-test.

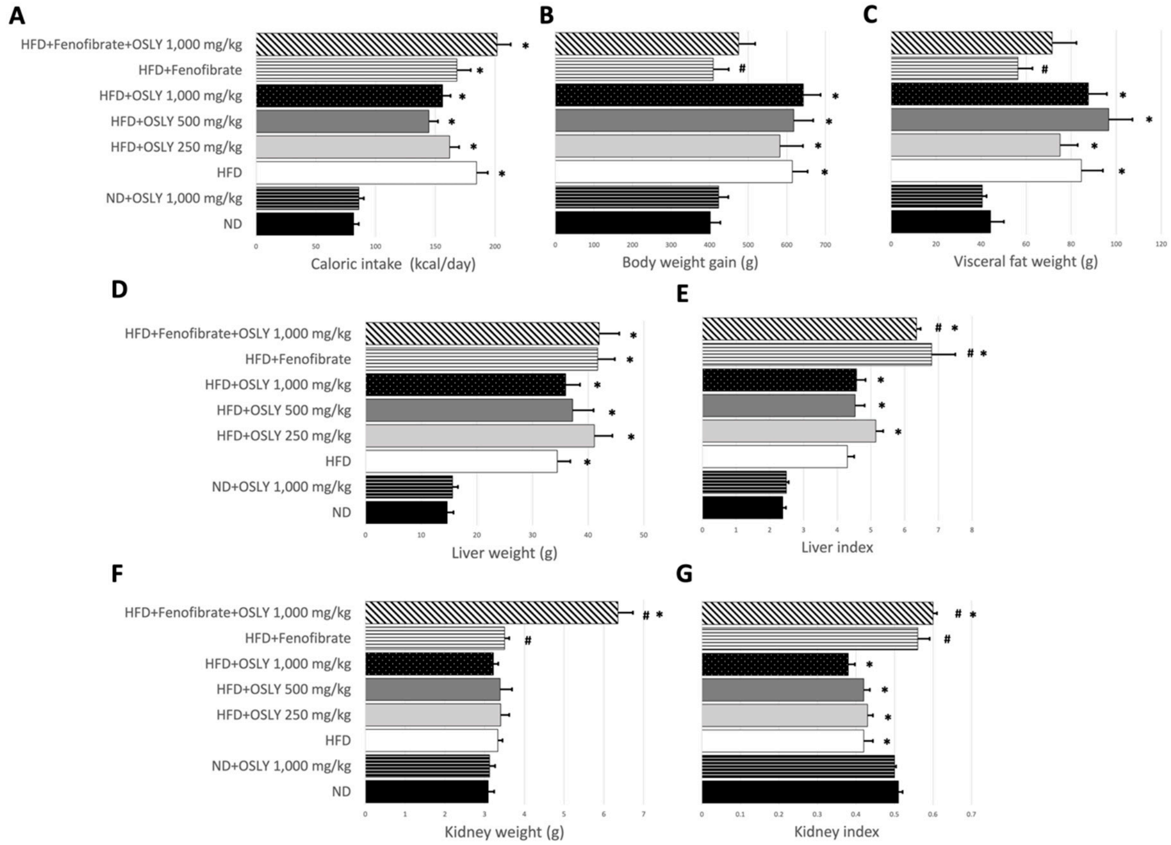

Figure S2. Serum and hepatic lipid profiles in rats fed either a normal diet (ND) or a high-fat diet (HFD). The rats were administered either vehicle (distilled water), *Ocimum sanctum* L. flower (OSLY) extracts at 250, 500, or 1,000 mg/kg body weight, and/or fenofibrate daily for 12 weeks. *Indicates significant difference ($P<0.05$) from the ND group; #indicates a significant difference ($P<0.05$) from the HFD group based on t-test.

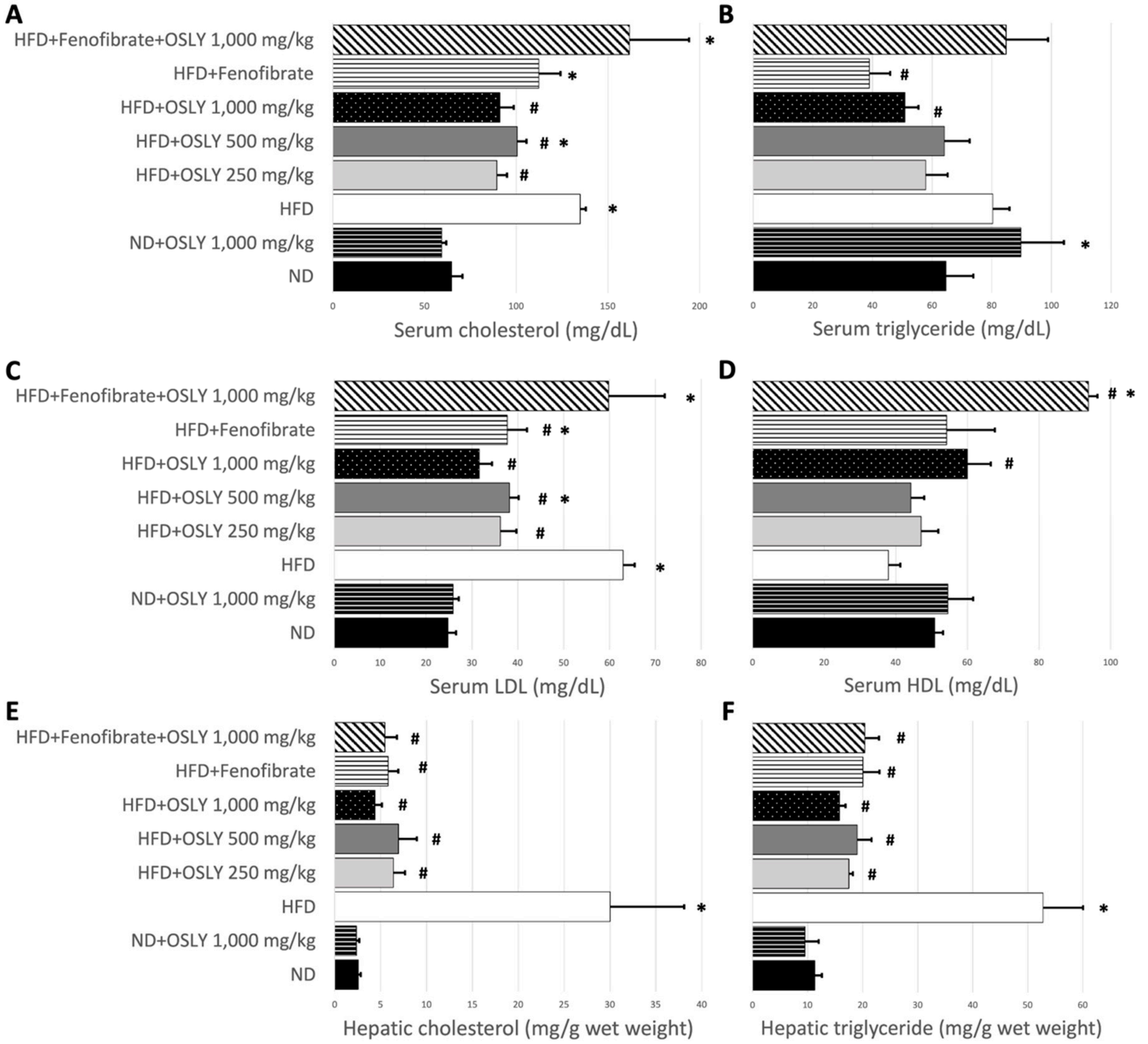

Figure S3. Serum biochemical markers of hepatic and renal functions in rats fed either a normal diet (ND) or a high-fat diet (HFD). The rats were administered either vehicle (distilled water), *Ocimum sanctum* L. flower (OSLY) extracts at 250, 500, or 1,000 mg/kg body weight, and/or fenofibrate daily for 12 weeks. *Indicates significant difference (P<0.05) from the ND group; #indicates a significant difference (P<0.05) from the HFD group based on t-test.

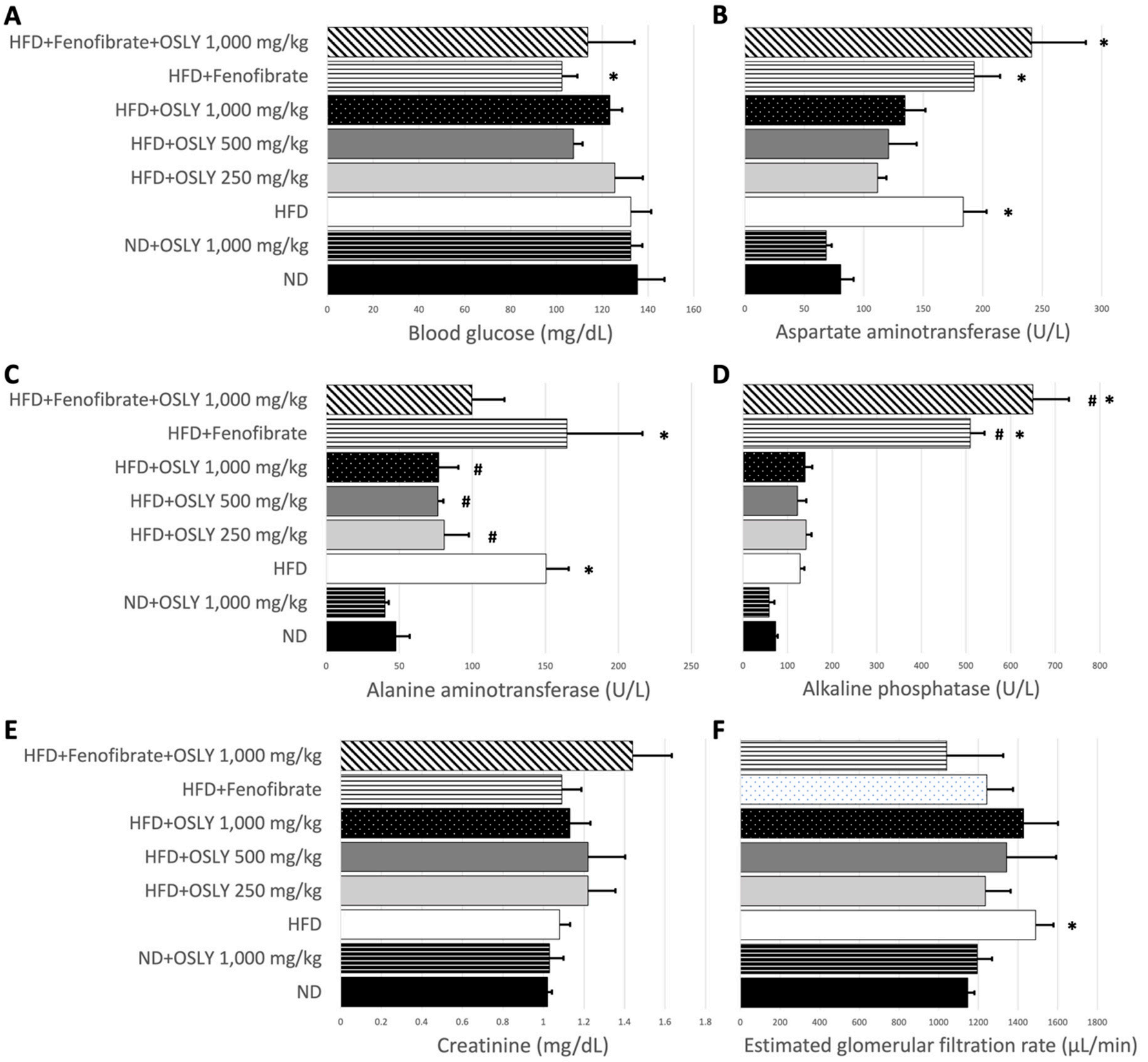

Figure S4. Hepatic and plasma malondialdehyde content in rats fed either a normal diet (ND) or a high-fat diet (HFD). The rats were administered either vehicle (distilled water), *Ocimum sanctum* L. flower (OSLY) extracts at 250, 500, or 1,000 mg/kg body weight, and/or fenofibrate daily for 12 weeks. *Indicates significant difference (P<0.05) from the ND group; #indicates a significant difference (P<0.05) from the HFD group based on t-test.

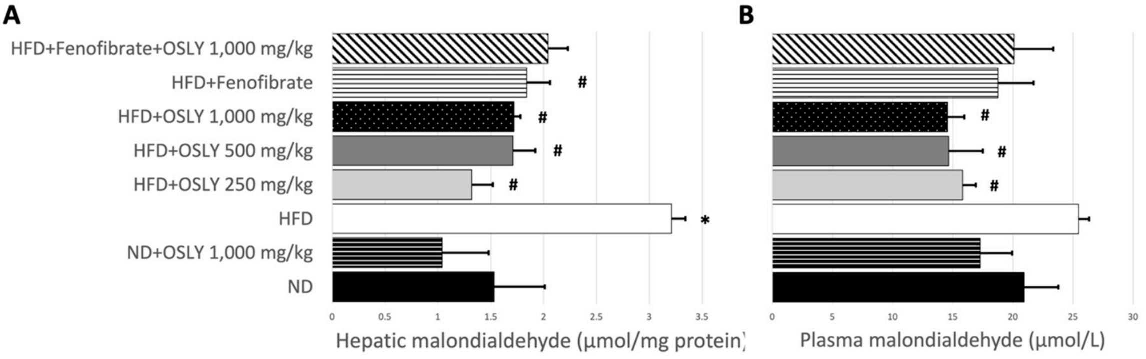

Figure S5. Principal component (PC) plot for hepatic choline metabolites. PC1 and 3 were associated with metabolic markers in the mediation analysis. Rats were fed either a normal diet (ND) or a high-fat diet (HFD). The rats were administered either vehicle (distilled water), *Ocimum sanctum* L. flower (OSLY) extracts at 250, 500, or 1,000 mg/kg body weight, and/or fenofibrate daily for 12 weeks. Abbreviations: Bet, betaine; Cho, choline, GPC, glycerophosphocholine, LPC, lysophosphatidylcholine; PC, phosphatidylcholine; Pcho, phosphocholine, SM, sphingomyelin.

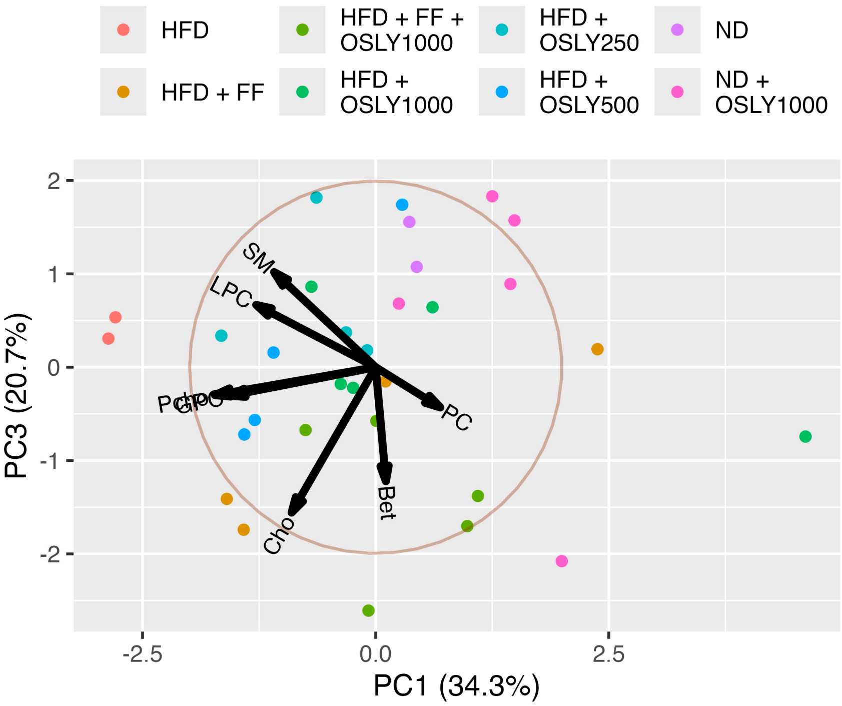

SUPPLEMENTAL TABLES

Table S1. Factor loading of each choline metabolite on principal components 1-3, which cumulatively explain 82% of the variance in the original variables.

	Principal component 1 (34%)	Principal component 2 (27%)	Principal component 3 (21%)
Betaine	0.04	0.52	-0.51
Choline	-0.29	0.14	-0.65
Phosphocholine	-0.56	-0.29	-0.12
Glycerophosphocholine	-0.51	-0.32	-0.13
Phosphatidylcholine	0.22	0.28	-0.18
Sphingomyelin	-0.35	0.46	0.42
Lysophosphatidylcholine	-0.41	0.48	0.28
